# Using spectral imaging for the analysis of abnormalities for colorectal cancer: When is it helpful?

**DOI:** 10.1371/journal.pone.0197431

**Published:** 2018-06-06

**Authors:** Ruqayya Awan, Somaya Al-Maadeed, Rafif Al-Saady

**Affiliations:** 1 Department of Computer Science and Engineering, Qatar University, Doha, Qatar; 2 Al-Ahli Hospital, Doha, Qatar; Universita degli Studi di Milano-Bicocca, ITALY

## Abstract

The spectral imaging technique has been shown to provide more discriminative information than the RGB images and has been proposed for a range of problems. There are many studies demonstrating its potential for the analysis of histopathology images for abnormality detection but there have been discrepancies among previous studies as well. Many multispectral based methods have been proposed for histopathology images but the significance of the use of whole multispectral cube versus a subset of bands or a single band is still arguable. We performed comprehensive analysis using individual bands and different subsets of bands to determine the effectiveness of spectral information for determining the anomaly in colorectal images. Our multispectral colorectal dataset consists of four classes, each represented by infra-red spectrum bands in addition to the visual spectrum bands. We performed our analysis of spectral imaging by stratifying the abnormalities using both spatial and spectral information. For our experiments, we used a combination of texture descriptors with an ensemble classification approach that performed best on our dataset. We applied our method to another dataset and got comparable results with those obtained using the state-of-the-art method and convolutional neural network based method. Moreover, we explored the relationship of the number of bands with the problem complexity and found that higher number of bands is required for a complex task to achieve improved performance. Our results demonstrate a synergy between infra-red and visual spectrum by improving the classification accuracy (by 6%) on incorporating the infra-red representation. We also highlight the importance of how the dataset should be divided into training and testing set for evaluating the histopathology image-based approaches, which has not been considered in previous studies on multispectral histopathology images.

## Introduction

Colorectal cancer is one of the most common cancers and the fourth most common cause of cancer death [1], with a high mortality rate in most of the developed countries. In 2012, it accounted for approximately 10% of the cancer cases recorded worldwide and found to be third and second most common cancer in men and women respectively [1]. The dramatic increase in the occurrence of cancer cases resulted in increasing demand for an automatic and objective measure that can reduce the subjectivity in assessment and act as a complementary tool for the pathologists. This can result in a provision of an effective treatment plan to the patients. Characterizing the abnormalities in colorectal tissue architecture and cellular morphology from biopsy slides plays an important role in deciding the treatment plan of the cancer patient or the patient with high risk of developing cancer in future. The correct identification of these abnormalities can reduce the risk of transformation of pre-cancerous tissue to cancer.

There has been a lot of work on developing automated systems for cancer detection as in [2–5] and more. However, most of these works are limited to the classification of only two categories (normal/benign and cancer) using RGB images and do not take other anomalies into account. There are few studies on colorectal cancer exploring the extended classes of benign [6, 7] and cancerous tissue [8, 9]. Further classification of cancer tissue into a low and high grade, as in above-mentioned papers, is important for deciding the treatment plan for the patient and also for determining the survival rate and so is the identification of different benign abnormalities for identifying the patients with high risk of developing cancer in future [10]. Keeping this in mind, we included images of two benign abnormality classes to our dataset along with normal and cancerous classes. Our multi-class classification consists of four classes: normal, small hyperplastic polyp (HP), tubular adenoma with low-grade dysplasia (TA_LG) and carcinoma (CA). The selection of these four classes is carried out in accordance with their pathological importance, details of which are given under *Methodology* section.

**Fig 1:**
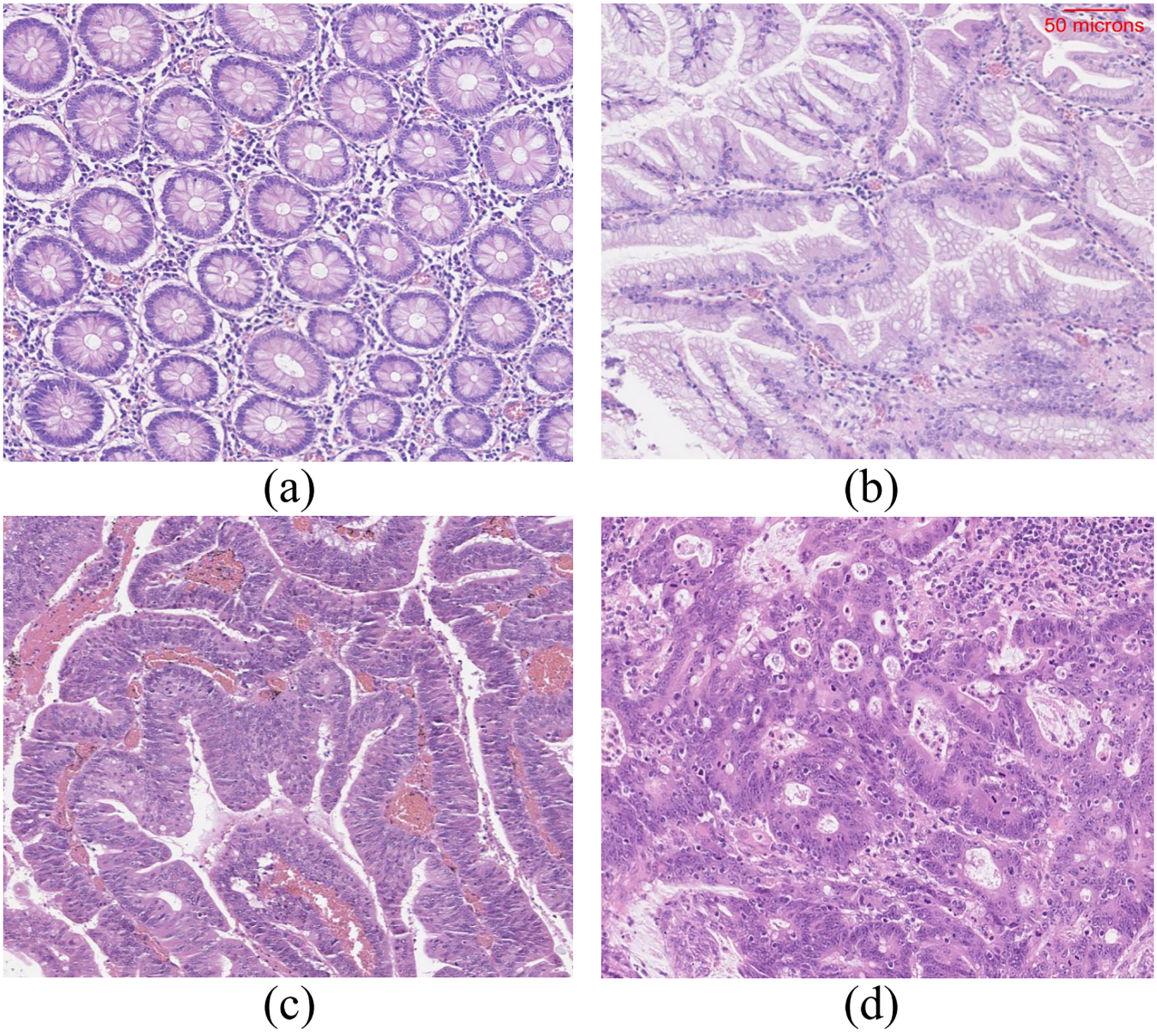
Example images of haematoxylin and eosin stained normal tissue and tissues with different abnormalities. (a) Normal tissue, (b) Hyperplastic polyp, (c) Tubular adenoma with low-grade dysplasia and (d) carcinoma. The scale bar shown on (b) is same for all the other sub-images. Note that these images are acquired using a different imaging system and are presented here to illustrate the tissue structure of different classes.

The glands of different tissue classes have varying textural appearance (see Fig 1). In normal tissue, the glands may appear either in the form of test tubes or in closed round shape depending on the cutting plane. In hyperplastic polyp, there will be well-formed, elongated glands and crypts with serrated (saw tooth) or star-shaped glands. In carcinoma, the glands may appear to be well/moderately/poorly differentiated or undifferentiated. Therefore, a texture feature based approach would be ideal for the stratification of these tissue classes. However, a single texture descriptor does not have the ability to capture the high variability in textures due to the fact that the abnormality of one class tends to have visual similarity with the abnormality of another class depending on their degree of aberrance. To cope with this variability issue, we use an ensemble of six different texture descriptors.

Many researchers considered the spectral imaging technique to have potential in the analysis of pathology images [11–13], in which each tissue section will be represented by a number of bands and wavelengths. Spectral imaging is based on constructing an image cube which comprises of a number of slices, representing images of the same scene captured with incident light of varying wavelengths. This imaging technique is known as multispectral imaging (MSI) if the resulting image cube comprises less than 40 numbers of slices; otherwise, it will be termed as hyperspectral imaging (HSI) [14]. Previous studies on histopathological analysis using multispectral imaging comprise of utilizing spectral information from visual spectrum (VS) only [3, 6, 7, 15–18]. In this study, we have included bands from infrared spectrum (IRS) which to the best of our knowledge is the first time to be used for the classification of colorectal images. In this paper, the term band is used for the 2D image captured at a specific wavelength. We evaluated the significance of adding IRS bands in a multispectral cube by comparing its ability to differentiate among classes with the ability of VS bands and with the combination of both VS and IRS bands. This comparison was performed for varying number of classes with different degree of complexity.

There have been discrepancies among previous studies ([16, 17]) regarding the usefulness of spectral information for the classification of histopathology images. Keeping this in view, we analysed the ability of whole multispectral cube for anomaly identification in comparison with a single and small number of selected bands. We carried out this analysis for both easy and complex task. In an easy task, only two categories (normal and cancerous tissue) were considered for classification which is a fairly simple task since both normal and cancerous tissue appear significantly different from each other. While for the complex task, we performed multi-class classification to differentiate among normal, HP, TA_LG and CA. The addition of HP class increased the complexity because of its visual similarity with either normal or TA_LG category for some cases, depending on its degree of aberrance. Our results with both easy and complex classification revealed the reason for not getting significant improvement in previous studies with the whole multispectral cubes in comparison with a single band or subset of bands.

There has been some work on multi-texture based classification ([6, 7, 19]) but contrary to our ensemble based learning, all these published methods follow single-classifier based learning with single texture feature or with various concatenated features. Using our dataset, we performed experiments with both these approaches to find which performs best. Using the best approach, we conducted the experiment on another dataset and found significant improvement. In an attempt to further improve the performance, we conducted global and pairwise feature selection using principal component analysis (PCA) and concave minimization.

The validation step is an important step for judging the classifier’s performance, specifically when the dataset is extracted from whole slide images (WSIs). In previous few studies on multispectral histopathology images, information related to the acquisition of images from how many different patient’s slides is not provided. This raises a question on the classifier’s performance on how it will perform on a new patients’ data. In this work, we present a detailed analysis on how much the data split criteria affects the classifier’s performance and report similar findings as in [15].

This study offers following contributions: 1) inclusion of tissue classes which are pathologically significant and are prone to developing cancer, 2) employing ensemble of classifiers, each trained with a different texture feature to cope with the challenges posed by pre-cancerous classes in this study, 3) inclusion and evaluation of infrared spectrum bands for the first time, 4) analysis of discrepancies among previous studies by performing 2-class and 4-class classification, 5) experimenting with global and pairwise feature selection to study its effect on the performance of multispectral image classification and 6) evaluating our approach using both weak and strong cross validation to justify the importance of splitting the dataset into training and testing set at patient level.

The rest of the paper is organized as follows: Section 2 gives a short background and related work on spectral imaging and its use for the analysis of histopathological data. Section 3 provides a brief discussion on the selection of four classes of tissue. It also presents our proposed methodology for feature extraction, dimensionality reduction, band selection and classification. Section 4 describes the experimental material and setup and presents a discussion on our findings. Section 5 presents concluding remarks and discusses the future work.

## Related work

MSI and HSI have been used for remote sensing applications for over a number of years. The use of this imaging for the computational analysis of pathologic tissues dates back more than 60 years [20]. In [20], infrared spectroscopy was used to investigate the molecular composition of normal and neoplastic tissue. In [21], infrared spectroscopic imaging is proposed as a replacement for tissue enhancing staining techniques to study different tissue components. The infrared spectral analyses were used to differentiate the malignant prostate tissue from the benign tissue sample. In most of the previous studies on the analysis of multispectral histopathology images, various morphological and texture-based features have been used for their classification. In [22], ten different morphological features describing the shape, orientation and other geometrical attributes of the nuclei were used to differentiate between normal and malignant image of colon tissue. In [17, 23], circular local binary pattern (cLBP) features were used generate the textural representation of benign and malignant colorectal tissue samples. In [6], various texture based descriptors were evaluated for the classification of colorectal tissue samples using MSI; and LBP, when calculating at multiple scales, was found to outperform other texture descriptors. The LBP features were calculated for each band separately and were concatenated together to characterize the whole multispectral image. In their later study [24], the authors proposed a multispectral multi-scale LBP (MMLBP) to integrate the spectral dimensionality of MSI which outperformed their previous approach in [6].

Glands are known to be the effective indicators for determining the extent of abnormality in case of adenoma and adenocarcinoma. To evaluate the efficacy of glandular features in stratifying three different types of tissues (benign hyperplasia, intraepithelial neoplasia and carcinoma), features were extracted from the segmented images [7, 25]. The segmentation was performed using the snake algorithm followed by feature extraction using gray-level co-occurrence matrix (GLCM), the Laplacian of Gaussian (LoG) and discrete wavelet transform. In [26], convolutional neural network (CNN) was employed to learn the hierarchical features from the gland segmented multispectral colorectal images. In [27], authors presented a comparison between MSI and RGB images by performing three class classification using a combination of texture and morphological features extracted from the gland nuclei. Their comparison results demonstrate that the classification accuracy is improved on utilizing the multispectral information when compared with the features extracted from the RGB images. In [28], an extension of GLCM texture features is proposed to capture the joint texture information along the spectral bands for prostate cancer diagnoses.

There is a number of other studies exploring the benefits of spectral imaging for the analysis of histopathology images, with some presenting promising results. In [15], the authors have shown the potential of HSI for identifying cancerous tissue taken from colon biopsies with high discrimination rate. In [18, 29], MSI is being evaluated for the detection of mitotic nuclei and for the segmentation of nuclei. However, the full benefit of using spectral imaging for the analysis of histopathology images is yet to be explored; for instance, the study of correlation between the light spectrum at different wavelengths and the type of tissues can open new directions. In two studies [16, 17], it was demonstrated that the spatial information is enough to capture the discriminatory information from the histopathology images and the additional spectral information does not add much information to improve the classifier accuracy. This may be due to a number of reasons. Firstly, in these studies, all the bands were captured using visible part of light spectrum. While it is assumed that the images captured using infrared part of the light spectrum may enhance the significant characteristics of different types of tissue. Keeping this in mind, we have captured images using infrared part of light spectrum along with the visible light spectrum. Secondly, there is a possibility that the bands at a certain wavelength may not be distinctive enough, thus reducing the overall performance of these images. It could be because of the staining characteristics as it may affect the resultant MSI/HSI. Thirdly, the classification task performed in these papers is focused on distinguishing between normal and cancerous tissue which is fairly a simple task. It might be the reason that they have found comparable results with the single bands and with the whole spectral cubes. To study the relation of a number of bands required in relation to the complexity of the task at hand, we have performed experiments with both 2-class (Normal vs Cancerous (CA)) and 4-class classification (Normal vs HP vs TA_LG vs CA).

## Materials and methods

**Fig 2:**
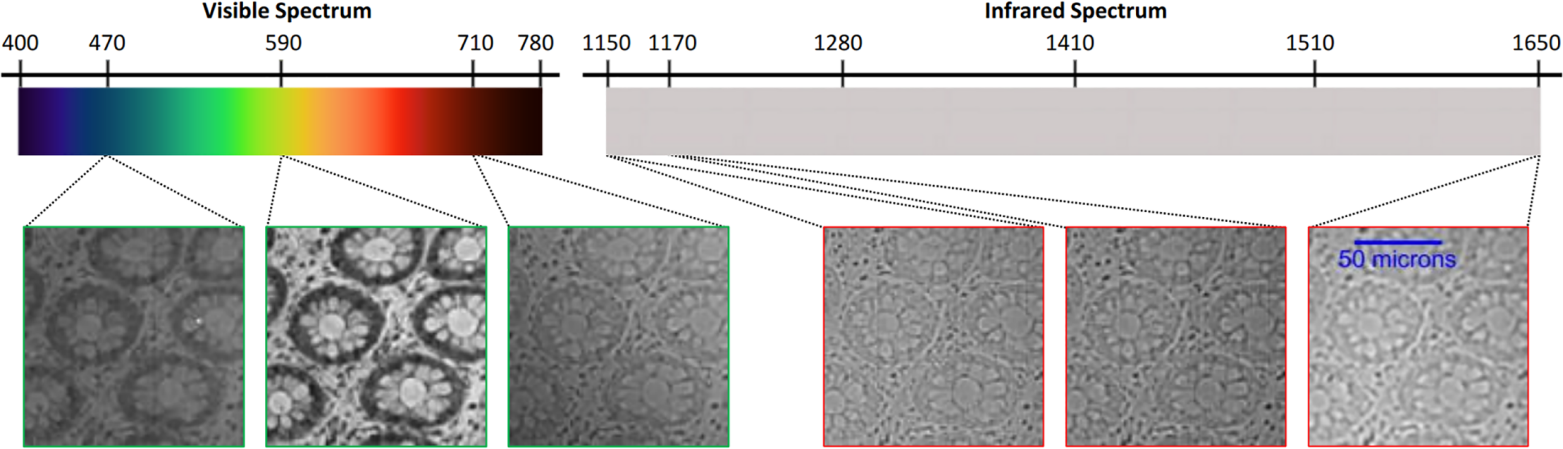
Slices/bands of a multispectral image cube of a normal tissue captured using light spectrum of different wavelengths. First 3 slices with green boundary are captured using visible part of the light spectrum with central wavelength [470 590 710]. Next 3 slices with red boundary are captured using the infrared part of light spectrum with central wavelength [1150 1170 1650]. The scale bar shown on the rightmost slice is same for all the other slices.

This study was reviewed and approved by the Qatar University’s Institutional Review Board (QU-IRB) and Al-Ahli Hospital Ethical committee which includes Medical Director and members from the different departments (Internal Medicine, General Surgeon and Ophthalmology) of Al-Ahli Hospital before the study began. The colorectal biopsy tissue slides were obtained from the Pathology and Laboratory Medicine lab at Al-Ahli hospital, Qatar. These tissue samples were fixed overnight in 10% neutral buffered formalin. Later, using the auto-processor, tissue samples were subjected to different concentrations of ethyl alcohol, then xylene and embedded in paraffin. The paraffin-embedded tissue was cut by microtom into 5 micron thickness, mounted on a glass slide and finally stained by Haematoxylin and Eosin (H&E) stain. The data acquired belonged to 151 different patients. The data was provided after de-identification and informed patient consent was obtained from all subjects. For 141 patients, this dataset comprised one biopsy slide per patient while for remaining 10 patients, there were more than one slides (maximum 3 slides per patient). Thus the total dataset consisted of 164 biopsy slides, taken from the year 2007 to 2016. The multispectral image dataset was captured at magnification power 10×, consisting of 200 multispectral images of size 256×320 in spatial dimensions with 39 spectral dimensions. These images are formed via light transmission through a tissue sample using a microscope which are then captured with a camera mounted over the microscope. Details of this dataset are provided in Table 1. Our image acquisition system consisted of four main components, Zeiss Axioscope A1 optical microscope with halogen source for transmission, a Xenics Camera and two tunable filters for multispectral imaging. The multispectral filter details are: Varispec Model VIS–20–20 filter with wavelength range 400 − 720nm to capture bands using VS and Varispec Model LNIR–20–20 filter with wavelength range 850 − 1800nm to capture bands using IRS. The bandwidth of these filters was 20nm. To capture the VS bands, a VIS filter was fitted over the microscope which was equipped with a Xenics camera (top port) to capture the visual appearance of tissue at specific wavelengths. This filter was replaced with LNIR filter in order to capture tissue at different wavelengths within IR range. We considered a range of wavelengths to capture physiological characteristics of tissues. 13 bands were sampled uniformly using VS across the wavelength of range 470 − 710nm and 26 bands were sampled uniformly using IRS across the wavelength of range 1150 − 1650nm. All these bands are spaced 20nm apart. Fig 2 shows few bands taken from both VS and IRS. Our dataset is labelled by an expert pathologist and comprises 50 normal, 50 HP, 50 TA_LG and 50 CA images. Each image is divided into four images of size 64×80×39 and is given the same label as the image from which they are extracted. This image division does not only generate more samples for training and testing but also allows us to generate images with varying texture information.

**Table 1:** Colorectal dataset details. Our experiments were conducted on 200 images taken from 151 patients. For some patients, our dataset comprises more than one biopsy slide.

	Normal	HP	TA_LG	CA	Total
No. of images extracted	50	50	50	50	**200**
No. of patients	31	36	38	49	**151**
No. of biopsy slides	38	38	38	50	**164**

For strong cross validation, the dataset was split into three folds such that all the images of a patient’s biopsy slide/s are included in one fold only while keeping an equal number of images for each class in an each fold. The dataset split into train and test set was carried out randomly based on patient’s unique case number. All the images of any selected patient were included in the fold if they satisfy the class balance. To assess the predictive performance of strong cross validation, the classification was performed three times and for each experiment, one part of data split was taken in test set while other two sets were taken in training set. While for weak cross validation, 3-cross validation was performed on image-level rather than patient-level, resulting in images from the same patient to appear in both training and testing set.

### Selection of multiple classes of tissue

In case of cancer being diagnosed, its grade is one of the factors that the treatment plan team takes into account. On the other hand, if a patient is cancer free but the biopsy slides show abnormalities in tissue architecture, then there is a need to stratify them in accordance with their degree of aberrance. The degree of aberrance such as in HP and adenoma with low and high grade dysplasia can be used to predict the risk of developing cancer in future. Small classic distally located HP is usually considered benign and is very unlikely to progress to carcinoma. On the other hand, adenomatous dysplastic polyps behave differently and are irreversible and can make its way to form carcinoma. Therefore, patients diagnosed with dysplastic adenomatous polyp have to be followed up to ensure removal of the whole polyp. Identifying these abnormalities is important since there is a possibility of progression from no dysplasia (either normal or hyperplastic polyp) to low grade dysplasia to high grade dysplasia to carcinoma [10]. Keeping this in mind, we collected a dataset comprising normal, HP, TA_LG and CA classes. However, in clinical practice, cancer has also been diagnosed without proceeding through each steps [30].

### Feature extraction

Inspired by Kather et al. work [19], we extracted texture features using six different texture descriptors. In their work, they analysed different features (histogram, perception, GLCM, LBP and Gabor) for classification of eight different classes using RGB images. We ignored Gabor filter for texture features because of its time complexity and neutral effect on accuracy, as in [19]. Rather, we experimented with two new features descriptors, LPQ and BSIF which are discussed below.

#### Haralick texture features

These texture features are based on gray-level co-occurrence matrix (GLCM) [31, 32]. It takes the spatial relationship of pixels into account by considering the frequency of a pair of pixels (at a specific angle) with respect to their intensity values. In this work, we have considered a pair of pixels at five displacements from 1 to 5 pixels. To make it invariant to rotation, we computed GLCM for four particular angles (0, 45, 90 and 135) with all the spatial displacements, which resulted in 20 GLCMs. All the GLCMs computed for each displacement were then averaged to obtain a rotation invariant version. For each of five GLCM, four statistical measure was calculated: energy, contrast, homogeneity and correlation [31]. We obtained 20 × 39 = 780 features for each input image cube.

#### Histogram features

Histogram of intensity values can be used to describe the texture of an image [32, 33]. We calculated following features from the histogram of each slice in an image cube: mean, variance, skewness, kurtosis and a vector of central moments (from 5th to 11th), thereby obtaining 11 × 39 = 429 features for an input image cube.

#### Perception-like features

Our third feature set is based on the visual perception of an image [34]. In [35], Bianconi et al. used these features to discriminate between tumour epithelium and stroma. In our study, we computed five texture features: coarseness, directionality, line-likeliness, roughness and contrast. The resultant feature vector is of size 5 × 39 = 195 for each input image cube.

#### Local Binary Patterns (LBP)

Our fourth feature set was calculated using LBP. We worked with “16,2” configuration which uses 5 × 5 pixel block of an image with 16 neighbouring pixels present along a circle of radius 2 pixels. Each neighbouring pixel is assigned 1 if their intensity value is greater than centre pixel’s intensity; otherwise, 0 is assigned. This set of binary values is converted to a decimal number and is assigned to the centre pixel. The histogram values are then computed to generate a feature set. In this work, we reduced the resulting feature set by using rotationally invariant LBP proposed [36]. The calculated feature vector size per band is 4116 and for an image cube, the length of the resultant feature vector is 160524.

#### Local Pattern Quantization (LPQ)

LPQ was initially presented as a texture descriptor which is robust to blurring effect [37]. Since this texture descriptor uses low frequency phase component, therefore it is also invariant to changes in illumination. The process of feature extraction using a window of fixed size and the binning of these features is similar to LBP. Although, our dataset doesn’t comprise of blurry images but we used this method as it extracts features based on different criteria and also it has been shown to perform better than LBP in non-blur images [38]. In this study, we have used its rotation invariant version of LPQ as proposed by Ojansivu et al. [39]. We computed LPQ features with a window size of 15 pixels and across 36 angles. The resultant LPQ feature vector is of length 9984 (256 per band).

#### Binarized Statistical Image Features (BSIF)

This texture descriptor is inspired by the LBP and LPQ [40]. Similar to LBP and LPQ, this feature set gives histogram based representation for an image. Instead of using predefined filters, it uses filters that are learned from the statistics of natural images, as provided by the authors of [41]. The statistical independence among the filter’s output is important to make sure that each filter extracts important but different features from other filters in a set. This filter independence was achieved by using independent component analysis (ICA) and principal component analysis (PCA). The BSIF approach takes two parameters: 1) size of the filter and 2) number of filters. The invariance to rotation is important for classification of images containing glandular structures since glands can appear in a number of orientation. The drawback of using BSIF is that it doesn’t possess rotation invariance property. We used 10 set of filters of window size 7 pixels. These filters were learned using a small set of natural images [40]. The resultant BSIF feature vector is of length 39936 (1024 per band).

### Band selection

In order to prove that the multispectral cube may have more information to improve the classification accuracy as compared to a single band or collection of few bands, we first trained our classifier with each band’s feature set separately. We found that the single band doesn’t contain enough information to provide results comparable to the result of a classifier trained with all the bands. Next step was to check if there may be a small size set of bands capturing enough information as that of a whole cube. To check with all the possible combination of bands was computationally expensive. Therefore, we adopted a similarity-based band selection approach known as linear prediction (LP) as proposed in [42]. A brief explanation of this approach is given in this paper and for further details reader is referred to [42]. The main steps of the method given in [42] are: 1) Removal of bad band 2) Image whitening 3) Selecting an initial pair of bands 4) Further band selection based on a similarity based criteria.

#### Removal of bad band

In [42], authors followed this step to remove noisy bands which may appear to be distinct from the rest of the bands but may be less informative. They computed spectral correlation coefficients between adjacent bands and removed those with low correlation coefficients. Since our dataset does not contain noise, therefore the computed correlation coefficients were observed to be greater than 0.8 except for the two adjacent bands, one belonging to VS and other belonging to IRS. The resultant value for these bands was low since they belong to two different light spectrums and cannot be considered as a bad band for removal. Considering this, we didn’t perform this pre-processing step on our dataset.

#### Image whitening

In [42], it is showed that the LP method performs better when used with the whitened bands. We performed whitening on a given set *X* = (*x*_1_, *x*_2_, …, *x*_*m*_), containing *m* number of multispectral images of dimension [*r* × *c* × *b*], where *r*, *c* and *b* represent number of rows, columns and bands respectively. To perform whitening, we transformed *X* into $X^{{}^{\prime }}=\left(X_{1}^{{}^{\prime }},X_{2}^{{}^{\prime }},\ldots ,X_{b}^{{}^{\prime }}\right)$ where each element of *X*′, let’s say, $X_{i}^{{}^{\prime }}=\left(x_{1i},x_{2i},...,x_{mi}\right)$ contains all the pixel values of a band *i* from all the m samples and is of dimension [(*r* * *c*) × *m*]. The whitened band *i* for all the samples can be obtained by $X_{i}^{{}^{\prime \prime }}=WX_{i}^{{}^{\prime }}$, where *W* is a whitening matrix. There are many choices for whitening matrix such as ZCA, PCA and Cholesky whitening matrices. In this work, we have experimented with PCA (λ^−1/2^*U*^*T*^) whitening mask where λ is the diagonal matrix of eigenvalues and *U* is the orthogonal matrix of eigenvectors.

#### Selection of initial pair of bands

The initialization of algorithm for LP requires selection of a pair of bands with the highest dissimilarity. The selection of these two bands is critical and affects the performance of the LP. Since our dataset comprises of bands from two different light spectrums, therefore we selected one band from the middle of VS and another band from the middle of IRS.

#### Linear prediction based band selection

LP method uses initial pair of bands *S* = (*B*_1_, *B*_2_) to generate a prediction for the next band *B*_*pred*_. Each element of *S* contains pixel values of the selected band taken from all the multispectral samples. An original band *B*_*orig*_ from the list of bands would be included in *S* if *B*_*pred*_ − *B*_*orig*_ is maximum as compared to the other bands. For every other inclusion of band, the size of *S* would increase and a number of bands in *S* will contribute to generate a *B*_*pred*_. *B*_*pred*_ will be generated using a set of parameters (*a*_0_, *a*_1_, …, *a*_*s*_) to minimize the prediction error and is formulated as follows:

$$\begin{array}{c} B_{pred}=a_{0}+\sum_{j=1}^{s}a_{j}B_{j} \end{array}$$ (1)

where *B*_*j*_ belongs to *S* and s is the number of bands in *S*. The set of parameters (*a*_0_, *a*_1_, …, *a*_*s*_) are estimated by

$$\begin{array}{c} a={\left(Y^{T}Y\right)}^{-1}Y^{T}y \end{array}$$ (2)

where *Y* is a matrix of size [*n* × (*s* + 1)] with *n* representing the number of pixels in a band. The first column of *Y* contains all ones while every other column contains pixel values of each selected band from *S*. *y* is the *B*_*orig*_ band flattened to form a column vector.

In [42], the authors have demonstrated that the small subset of pixels in most cases produces same results as compared to using all the pixels. Hence, we performed band selection using patches of size 64 × 64 pixels extracted from the middle of each band.

### Pairwise vs global dimensionality reduction

Dimensionality reduction reduces the computational complexity and storage requirement and is more likely to improve the classifier performance by the selection of uncorrelated multi-class feature subset [43]. Dimensionality reduction approaches can be broadly categorized into feature selection and feature extraction. Feature selection methods use some criteria to select a subset of features while feature extraction approaches transform the original feature set into a modified feature set with more relevant feature information [44]. Feature selection methods can be further classified into three categories: filter, wrapper and embedded [45]. The main focus of performing this experiment is to evaluate pairwise and global multi-class feature reduction for improving the classifier’s ability to learn.

For a multi-class classification problem, SVM classifier performs all possible combination of either one-vs-all or one-vs-one binary classification. Most often, a single feature set is selected for all the combination of binary classifiers, which we refer as global feature reduction in this paper. There is a possibility that the features which perform best for one pair of classes may not perform well for another pair of classes. In [46, 47], authors have shown that pairwise feature selection performs better than global feature selection, both in terms of accuracy and time. The overall flowchart for pairwise dimensionality reduction is shown in S1 Fig.

For our experiments, we opted one widely used feature extraction method, PCA [48] and one wrapper approach to feature selection via concave minimization [49] using SVM. Our experiments have shown that PCA outperforms when used globally, even when a small number of features are extracted while the performance of pairwise FSV is better than global FSV but it requires a larger feature set for improved accuracy. Based on our experimental outcome, we used global PCA as our final feature reduction approach. The reader is referred to [50, 51] for details on global PCA based feature extraction. The results are shown in S2 Fig.

### Classification

**Fig 3:**
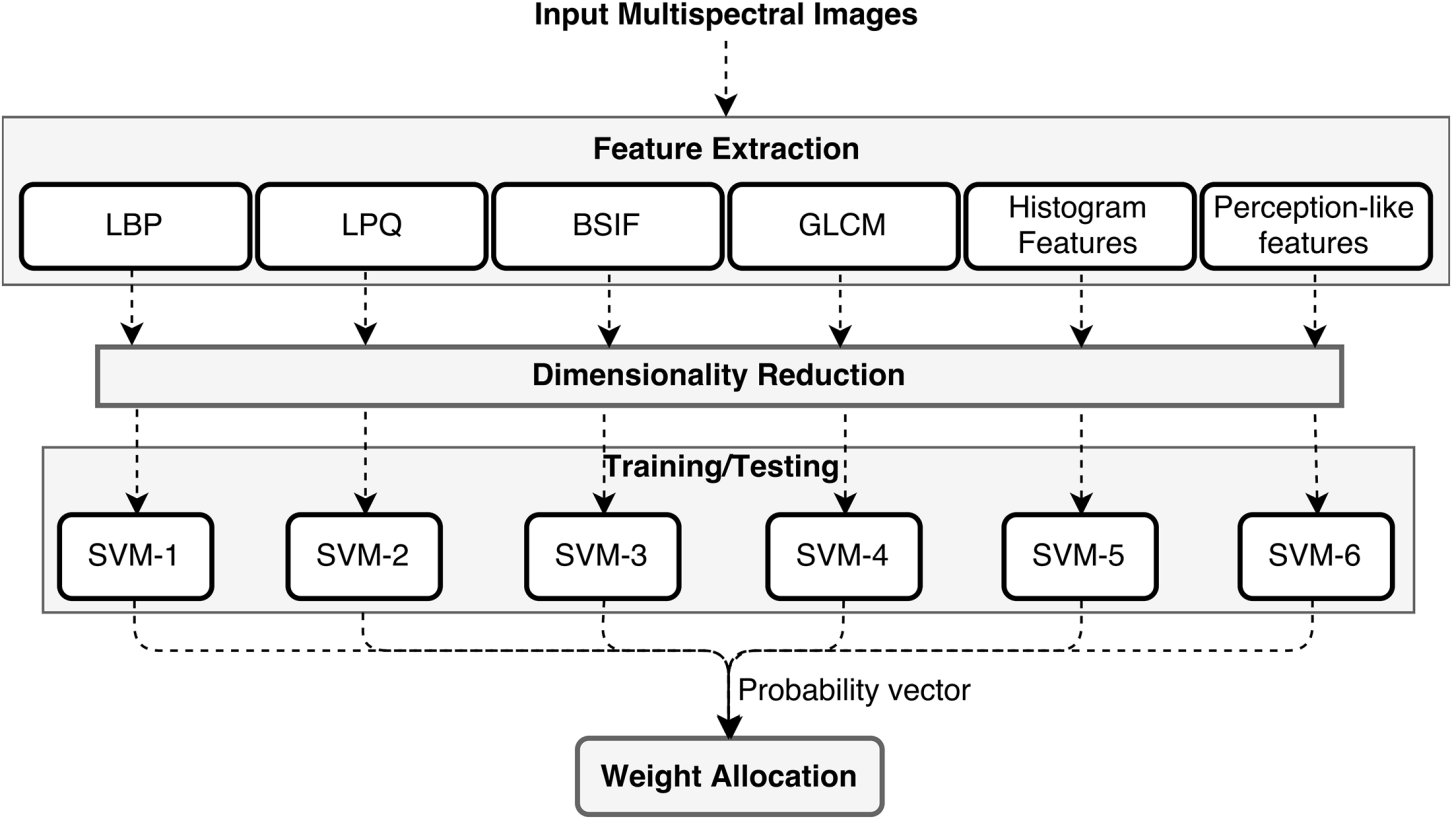
Overall flowchart of our proposed method. Each feature set is learned by a separate classifier. Weight allocation step represents training/testing a linear SVM classifier using the concatenated probability matrix of all the individual classifiers.

We followed two strategies for classification and proceeded with the best one with low error rate: 1) Feature concatenation approach: Train a single classifier on a concatenated feature set (combination of all features) 2) Ensemble learning approach: Learn each feature set using a separate classifier and give weightage to each classifier based on its ability to identify the correct label. In the second approach, weightage to each classier is given based on their classification probabilities on the training dataset. This was done by training a linear SVM classifier which we call it as global SVM on the resultant probabilities of each classifier. For testing, the concatenated probability vector of all the classifiers is given as an input to this global SVM. The overall flowchart of ensemble based classification is shown in Fig 3. For the first approach to classification, all the feature vectors were merged together, resulting in a very high dimensional feature vector. The feature reduction was performed after the concatenation. In the second approach, feature reduction was performed for each feature vector separately.

In order to study the effect of selecting training and testing patches from the whole slide images (WSIs) on classifier performance, we experimented with both weak and strong cross validation. In strong cross validation, the data split was carried out using patient’s data in such a way that if for any patient, his/her WSI is used for collecting training patches then testing patches would not be extracted from the WSIs of the same patient and vice versa. While in weak cross validation setting, no such conditions were taken care of. The goal of this comparison between strong and weak cross validation was to judge how our proposed method would perform outside the sample to a new patient dataset which our trained classifiers have never seen.

To study the correlation between complexity to distinguish between classes and the number of spectral bands required for classification, we have performed two types of classification: 2-class classification which represents discriminating between normal and cancerous tissues (CA) and 4-class classification which stands for classifying the images into normal, HP, TA_LG or CA.

## Results and discussion

**Fig 4:**
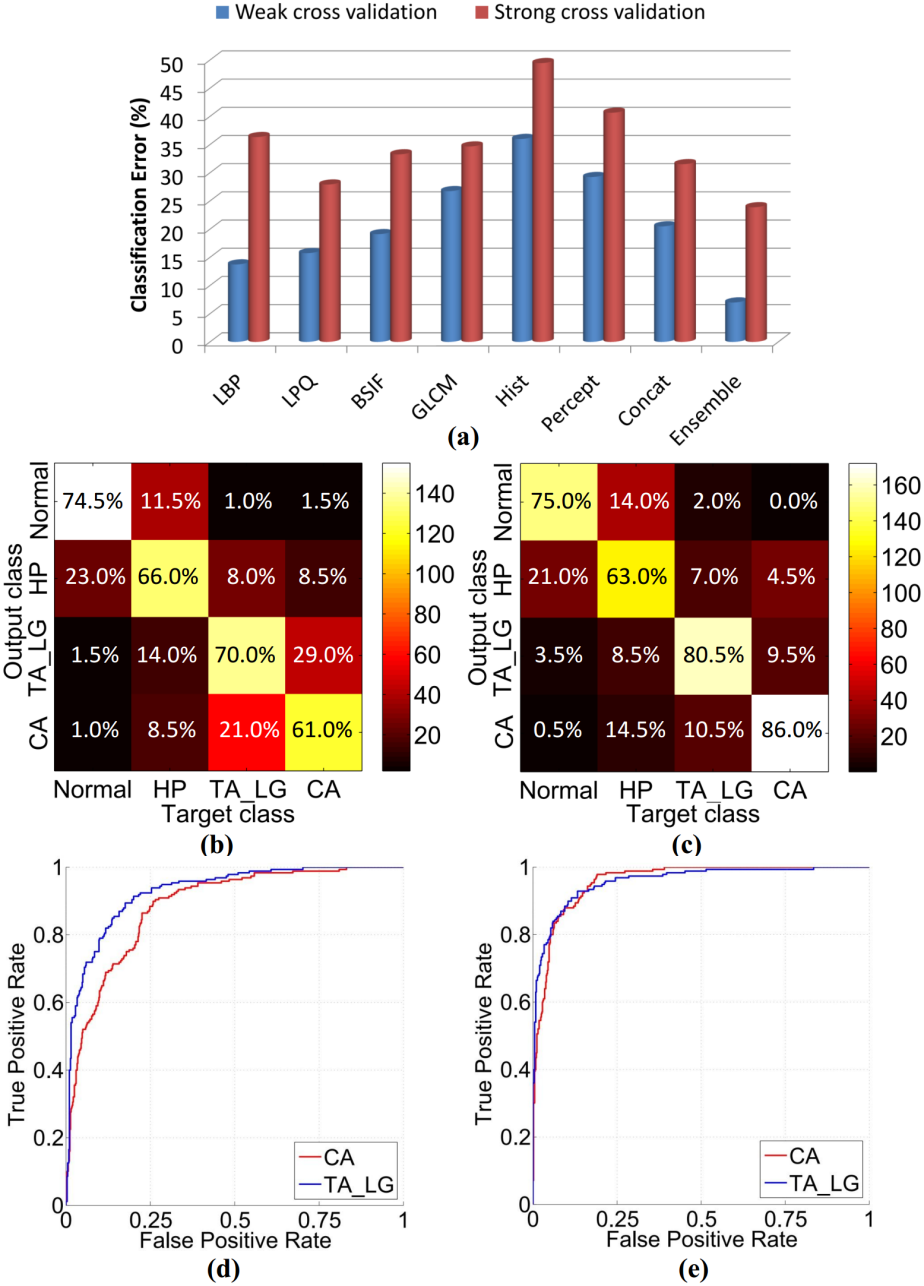
Performance comparison between concatenated and ensemble learning approach. (a) shows classification error (%) of classifiers when trained with various texture features (LBP, LPQ, BSIF, GLCM, Histogram and Perception-like features), concatenated feature set and ensemble learning. These results are presented with both weak and strong cross validation. (b) and (d) show confusion matrix and its corresponding ROC curves for the concatenated features respectively. The mean AUC for CA and TA_LG is 0.908. (c) and (e) show confusion matrix and its corresponding ROC curves for the ensemble learning respectively. The mean AUC for CA and TA_LG is 0.960.

The effect of weak and strong cross validation on classifier’s performance is shown in Fig 4(a). These results demonstrate that the split of data into training and testing patches has a significant effect on the classifier’s accuracy. It is due to the fact that the WSIs of the same patient would show similar morphology of both low and high level features (cell and glandular structures) while the WSIs of two different patients would show different histological architecture. Therefore, in case of weak cross validation, classifier performance is better as it has learned from similar images that will appear in the testing phase and would not guarantee the same performance on unseen data. This implies that the classifier’s true performance should be evaluated using strong cross evaluation on unseen patient data. Therefore, all the further experimental results using our own dataset are presented with strong cross validation, otherwise mentioned.

In order to improve the classification performance, we conducted experiments with our ensemble based learning and with the traditional feature concatenation approach. Our results demonstrate that our approach based on ensemble classification performs better as compared to the concatenated approach, in both weak and strong cross validation settings. The confusion matrices obtained using both these approaches are shown in Fig 4(b) and 4(c). HP class is observed to be the most complex class, due to its similarity with other classes. TA_LG and CA are considered as positive classes due to their pre-cancerous and cancerous nature and should be detected with high true positive rate (TPR). Two ROC curves are shown in Fig 4(d) and 4(e): 1) when only CA is considered as a positive class while for the second curve, CA class is excluded while considering TA_LG as a positive class. It can be seen from the results that the ensemble learning approach has brought significant improvement in TPR as compared to feature concatenation approach. Table 2 summarizes the performance of our ensemble based learning approach for two categories using strong and weak cross validation. In the first category, CA is considered as a positive class while in the second category, TA_LG, being an abnormality with a high risk of developing cancer, is also considered as a positive class along with the CA. The accuracy values reported in Table 2 represents the percentage of correctly predicted positive and negative samples.

**Table 2:** Summary of the ensemble based classification results. The reported values are *mean* ± *standard deviation*, where the variation is the result of cross-validation runs. Here accuracy refers to the percentage of correctly predicted positive and negative samples.

	Accuracy	Sensitivity	Specificity	F1-score
**Strong Cross Validation**				
CA	90.1 ± 0.5	86.1 ± 3.4	91.5 ± 0.6	81.3 ± 1.3
CA + TA_LG	89.9 ± 2.2	93.3 ± 4.8	86.5 ± 0.4	90.2 ± 2.4
**Weak Cross Validation**				
CA	96.1 ± 1.8	93.5 ± 4.6	97.0 ± 1.3	92.4 ± 3.5
CA + TA_LG	95.8 ± 0.4	97.3 ± 0.9	94.3 ± 0.9	95.8 ± 0.4

**Fig 5:**
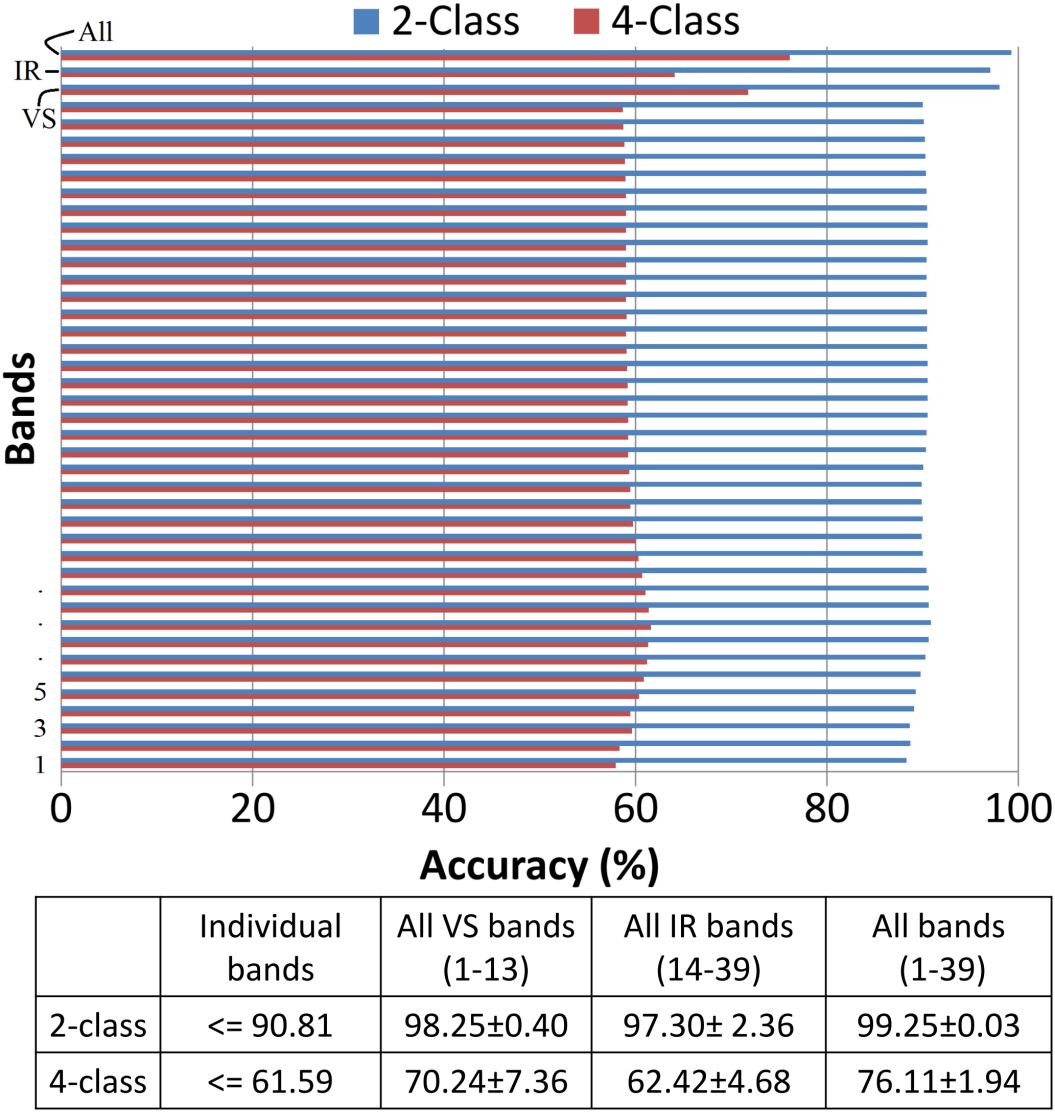
Classification accuracy of our method when trained with features extracted from individual bands, all VS bands, all IRS bands and all bands in a multispectral cube. For both 2-class and 4-class classification, classifier performs better when trained with features extracted from the whole cube in comparison to classifier trained with features from each individual band or all VS bands or all IRS bands. 2-class classification consists of normal and cancerous class only while 4-class classification has all the original classes.

To fully explore the true potency of multispectral information, we performed classification with each individual band, a combination of all VS bands, a combination of all IRS bands and combination of all the bands. Since IRS is used for the first time for classification of colorectal images, therefore we conducted this experiment to check if the IRS bands are contributing towards improving the results in combination with VS bands. The quantitative results of this experiment are shown in Fig 5. Our results demonstrate that the classifiers when trained with features obtained from VS bands perform better as compared to that of IRS bands. However, a combination of VS and IRS bands further improves the result. On the other hand, the accuracy obtained using individual band belonging to any spectrum was less than the accuracy obtained with any combination of spectrum bands. From this experiment, we conclude that multispectral cube contains additional information on comparing with individual bands.

**Fig 6:**
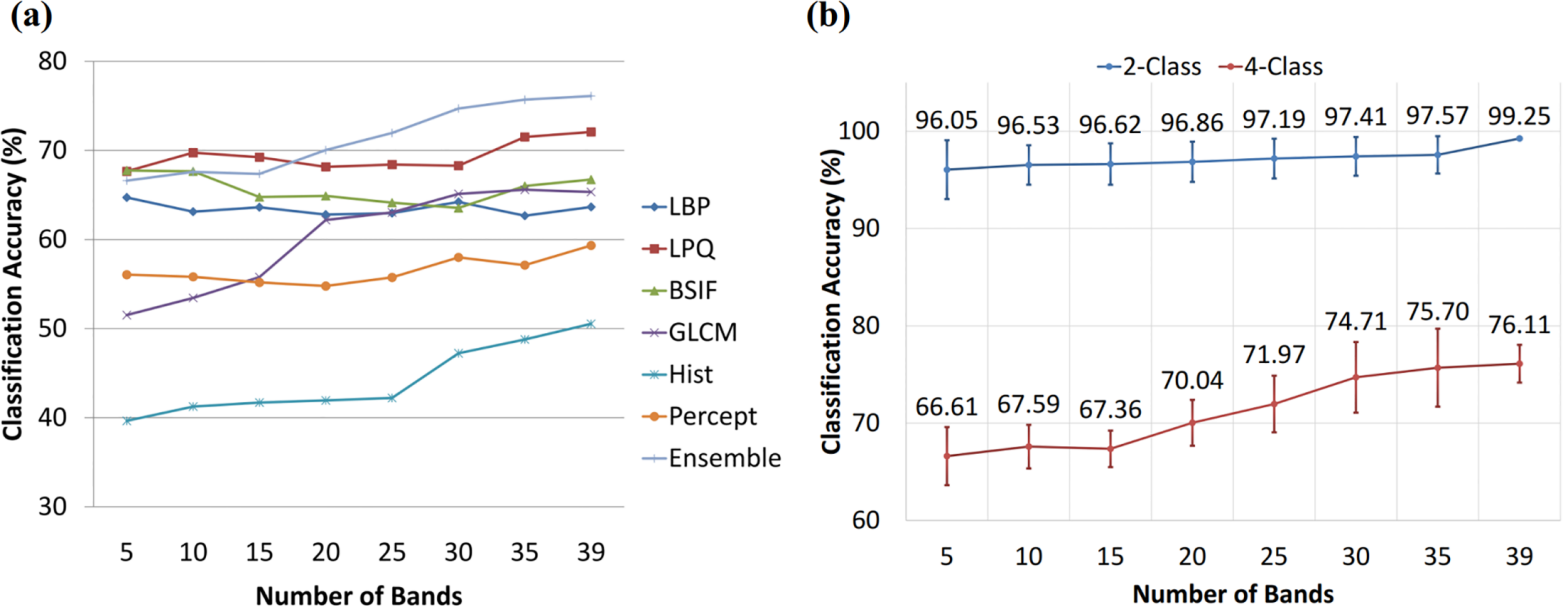
Classification accuracy of our method when trained with features extracted from varying number of selected bands. Various numbers of bands are selected using a method based on similarity based criteria, as described in [7]. (a) shows the results of selecting varying number of bands for each texture descriptor separately along with our ensemble approach (b) shows the results obtained for 2-class and 4-class classification. 2-class classification consists of normal and CA class while 4-class classification has all the original classes.

From the previous experiment, one can argue that there may be any combination of bands that could perform better or comparable to the whole multispectral cube. Conducting the experiment with all combination of bands was computational expensive, therefore we followed band selection criteria to extract the multiple number of bands [5,10,15,…,35]. With different texture descriptors, we observed different results as shown in Fig 6(a). These results are also presented in S3 Fig with error bars. It can be seen that classification improved on increasing number of bands with LPQ, GLCM, perception-based and histogram features but with LBP and BSIF, the classification accuracy for some combination of bands was better than with whole multispectral cube. LPQ was observed to be the most consistent feature descriptor in terms of performance across different cross validation folds (see S3 Fig). While our ensemble learning approach improved the accuracy on every addition of selective bands. Thus it is hard to make any judgement on the inefficacy of multispectral imaging based on any single texture descriptor. Previous studies, presenting results not in the favour of spectral imaging, have performed classification between normal and cancerous images. Therefore, in addition to our 4-class classification, we also performed 2-class (normal vs CA) classification. We observed that the degree of improvement in accuracy with respect to the number of bands is related to the complexity of the task at hand. In 2-class classification, since the task to differentiate between normal and CA is fairly easy, therefore there is only 3% improvement when using features from the whole cube as compared to the features from only 5 selective bands. While in 4-class classification, the task is difficult and we observed a significant improvement (10%). These results are shown in Fig 6(b). These results also demonstrate that for a simple task, the variation among different cross-validation folds is quite smaller when all the bands are used. While in case of using a different subset of bands, the variation is relatively high; although, it is uniform across different subsets. On the contrary, for the 4-class classification task, this pattern of variation is inconsistent due to the difficulty in discriminating normal and cancerous classes from the pre-cancerous classes.

**Fig 7:**
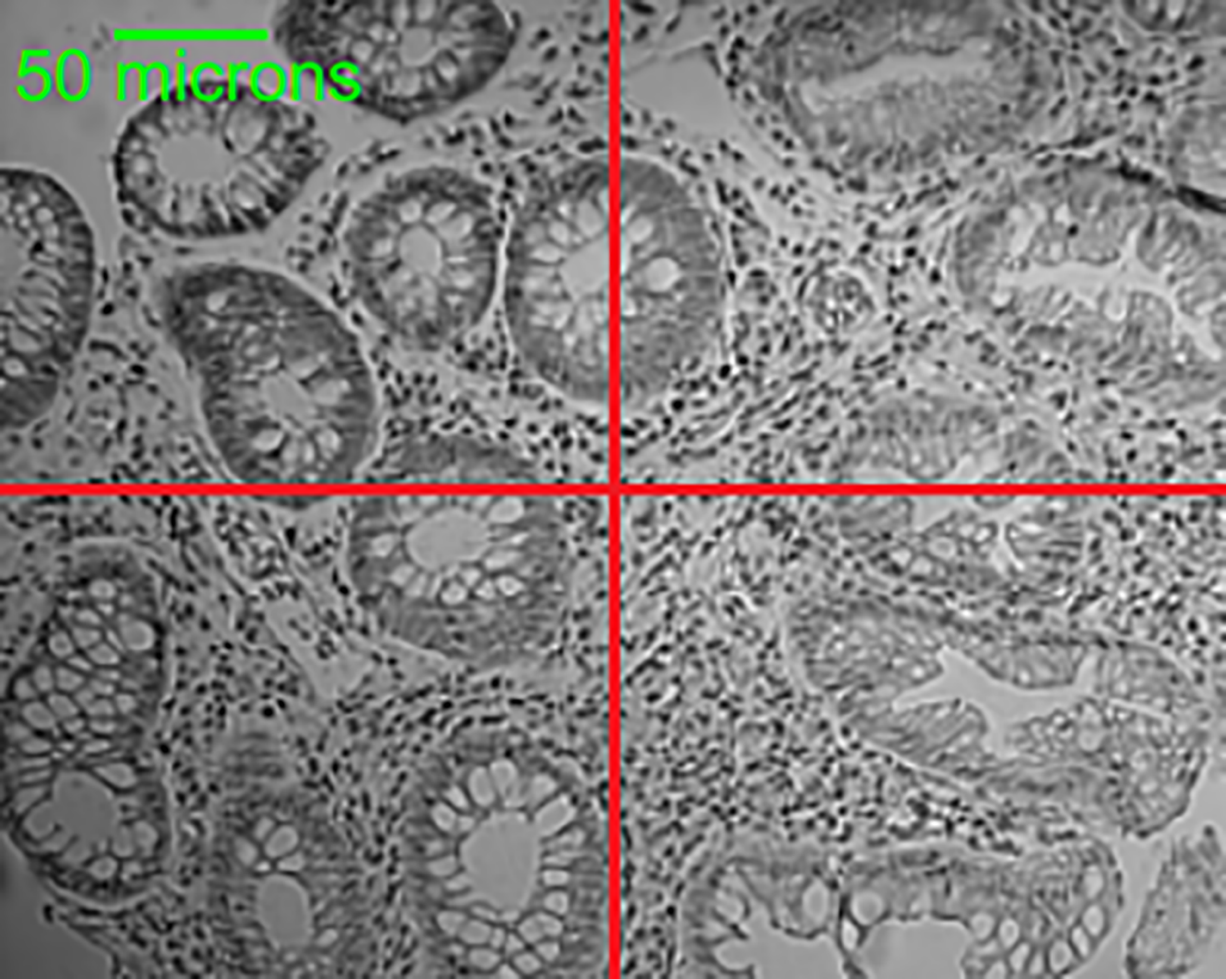
Single image showing tissue of two different labels. The ground truth label given by pathologist for this whole image is HP. While the sub-images on the left side contain normal tissue and sub-images on the right side comprise HP tissue.

The results reported in Fig 4 are computed for sub-images, instead of whole images for which labels are provided by the pathologist. The pathologist has assigned a label to whole images based on the highest degree of tissue abnormality i.e. if an extracted image contains both HP and TA_LG then TA_LG being more abnormal than HP would be assigned to the image. Our dataset contains such images (size 256×320 pixels) with some part of tissue belonging to one class and other part of tissue belonging to another class. An example image from our dataset is shown in Fig 7. This image contains both normal (left column) and HP (right column) glandular structures; since HP is an abnormal class therefore the pathologist has given it HP label. On splitting this image into four sub-images, the extracted sub-images show tissue belonging to only one class (either normal or HP). Our proposed system is trained on sub-images, each given a label of the whole image from which it is extracted. Hence, there are sub-images which may not have correct ground truth label for representing the tissues shown. Our method has identified these patches with the correct label which do not conform to the label of the whole image but are actually the correct identification of the tissue abnormality. The evaluation is performed against the pathologist assigned labels for whole images and is one of the reasons (along with small dataset) that our system has not performed well. For example, an image shown in Fig 7, our system has correctly identified the sub-images in the left column as normal and the right column sub-images as the HP but left column sub-images were considered misclassified since they were given the label (HP) of the whole image.

**Fig 8:**
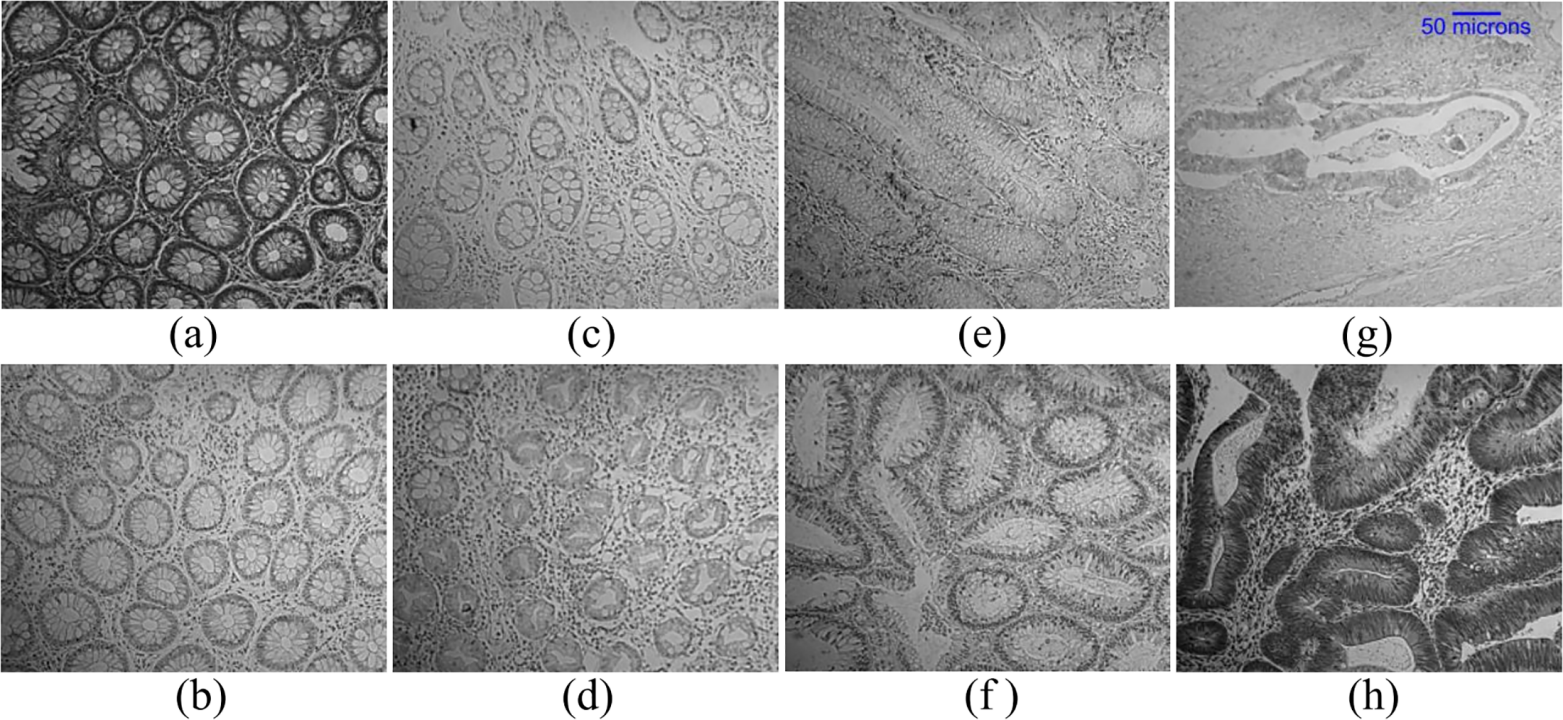
Test mages which are misclassified by our proposed method. (a) and (b) are labelled as normal by the pathologist but are misclassified as HP. (c) and (d) are labelled as HP and are misclassified as Normal. True label of (e) and (f) is TA_LG and are misclassified as CA and HP respectively. (g) and (h) are labelled as CA and are misclassified as TA_LG and HP respectively. The scale bar shown on (g) is same for all the other sub-images.

To evaluate the true performance of our system against the ground truth of whole images, we assigned final labels in a way that if there are sub-images with different predictions then the high occurring predicted label would be given to all the sub-images of a particular whole image. In case there are two sub-images predicted as one class and other two sub-images predicted as another class then the final label would be determined based on which label has the highest average probability. In this way, the accuracy using strong cross validation is increased from 76.1% to 81.4% for 4-class classification and for 2-class classification, accuracy is increased from 99.3% to 100%. Some example images of misclassification are shown in Fig 8.

### Comparison

The purpose of this comparative analysis is not to report the improvement in accuracy in comparison to the previously reported accuracies. It is to demonstrate that the classification approach followed in this study is appropriate enough for the assessment of multispectral imaging in terms of their suitability for histopathology analysis. The analysis of multi-textural descriptors and CNN features for medical spectral imaging (prostate and colorectal) has been investigated in previous studies. We have evaluated our multi-textural ensemble based classification approach with some recently published studies [6, 7, 24, 26] for which the dataset was available to us. This dataset consists of three classes: benign hyperplasia (BH), intraepithelial neoplasia (IN) and carcinoma (CA). The multispectral cube is formed by 16 bands captured using VS only. The comparison results are generated using 10-cross validation. We have presented our results on this dataset with two settings. In the first setting, parameters for features descriptors and classifiers fine-tuned on our multispectral dataset were used to generated results for this dataset. While in the second setting, these parameters were fine-tuned for this dataset. The comparative results demonstrate that our approach (with fine-tuned parameters for this dataset) has obtained comparative results when compared with the best-performing methods. These results in terms of accuracy are shown in Table 3, along with the features descriptors and classifiers used to obtain the corresponding accuracy.

**Table 3:** Results of ensemble learning approach and other comparison algorithms. LoG, DW and CNN represent Laplacian of Gaussian, discrete wavelet transform and convolutional neural network respectively. In *Our Approach 1*, parameters fine-tuned on our dataset were used while in *Our Approach 2*, parameters were fine-tuned for this dataset.

	Accuracy (%)	Feature Descriptor	Classification Model
Hassan et al. [26]	79.2	CNN features extracted from the unsegmented images	CNN
Peyret et al. [6]	88.3	Single-scale LBP	SVM
Peyret et al. [6]	91.3	Multi-scale LBP	SVM
**Our Approach 1**	**97.8**	[LBP,LPQ,GLCM,Hist, Perception, BSIF]	SVM
Chaddad et al. [7]	98.9	[LoG,DW,GLCM]	LDA
**Our Approach 2**	**99.1**	[LBP,LPQ,GLCM,Hist]	SVM
Hassan et al. [26]	99.2	CNN features extracted from the segmented images	CNN
Peyret et al. [24]	99.6	Stacked Multispectral Multi-scale LBP	SVM

## Conclusion

Since MSI has been observed to have more discriminative information than the RGB images therefore it is the focus of this study for multi-class classification of colorectal images. The scope of this paper is to analyse the usefulness of spectral information in order to address the discrepancies among previous studies. These discrepancies raise questions on the adoption of MSI for histopathology image analysis. In this paper, we carried out analysis of multispectral images of colorectal tissue, made up of slices obtained from both VS and IRS. The inclusion of IRS bands improved the accuracy by 6% for multi-class classification. We further demonstrated the importance of feature information along the spectral bands with respect to the complexity of data. Our results have shown that the use of whole multispectral cube brings significant improvement in TPR when the classes are hard to differentiate. Our comparative analysis of the two-class and the four-class classification demonstrates the reason of MSI not improving the classification accuracies in previous studies. The task in previous studies to differentiate between normal/benign and malignant images is a fairly easy task for which the spatial information is enough to discriminate between the two. However, to fully understand the reason of discrepancies among studies using MS/HP imaging, further research is required to study the correlation between the light spectrum at different wavelengths and the type of tissues under study. We also studied the importance of data split to judge the performance of a classifier and explained how strong cross validation is the valid approach for the evaluation of any classification approach. For classification, we used a number of texture-based image descriptors and based on our experimental findings, we suggest using ensemble learning rather than concatenating the feature set of all the descriptors. Although we performed multiple feature extraction independently, this means that the feature extraction procedure can potentially go parallel. It is still worth mentioning that multiple feature extraction is a time-intensive task for both training and testing phase. In future, we will extend this analysis on MSI by learning features from the data itself using the deep neural networks. Since the prerequisite to the deep neural network is the availability of large annotated dataset, hence our future work would also comprise further spectral image acquisition.

## Supporting information

S1 Fig Flowchart of pairwise dimensionality reduction method. In figure, *straight lines* refer to the training phase while the *dashed lines* refer to the testing phase. *C*_*i*_ represents features of class *i*.
(TIF)

S2 Fig Comparison between global and pairwise feature selection method. Comparison results are presented using two approaches: PCA and feature selection via concave minimization. Note that this experiment is performed using LPQ features with weak cross validation because of their low classification error.
(TIF)

S3 Fig Comparison of the results of different features. These results are generated by selecting varying number of bands for each texture descriptor. The error bars are generated as a result of cross validation.
(PNG)
