# Supplementary figures and images for: Using spectral imaging for the analysis of abnormalities for colorectal cancer: When is it helpful?
Source: PLoS One. 2018 Jun 6;13(6):e0197431. doi: 10.1371/journal.pone.0197431 (PMC5991384; doi:10.1371/journal.pone.0197431)

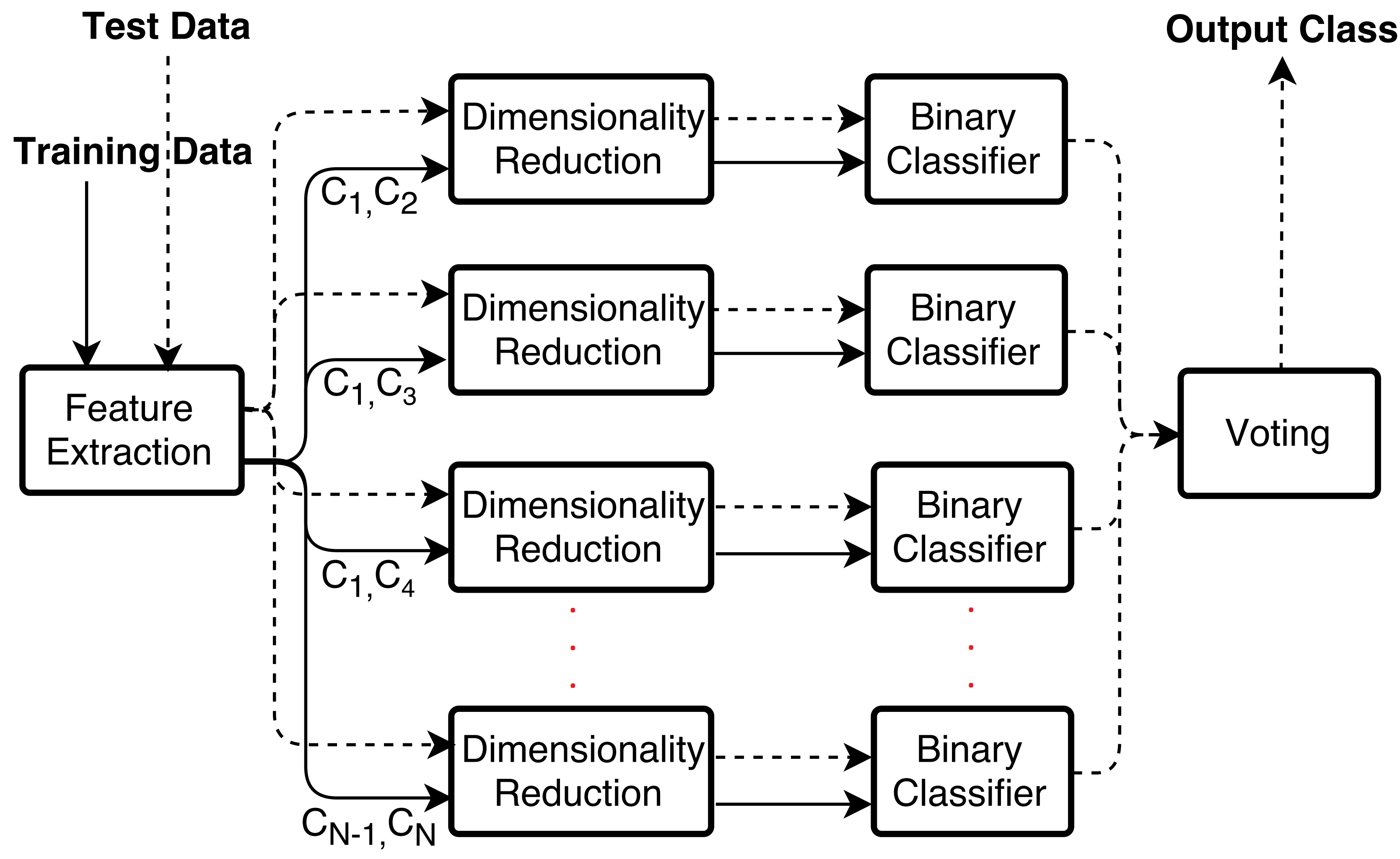

Supplement: S1 Fig — In figure, straight lines refer to the training phase while the dashed lines refer to the testing phase. Ci represents features of class i. (TIF) [file pone.0197431.s001.tif]

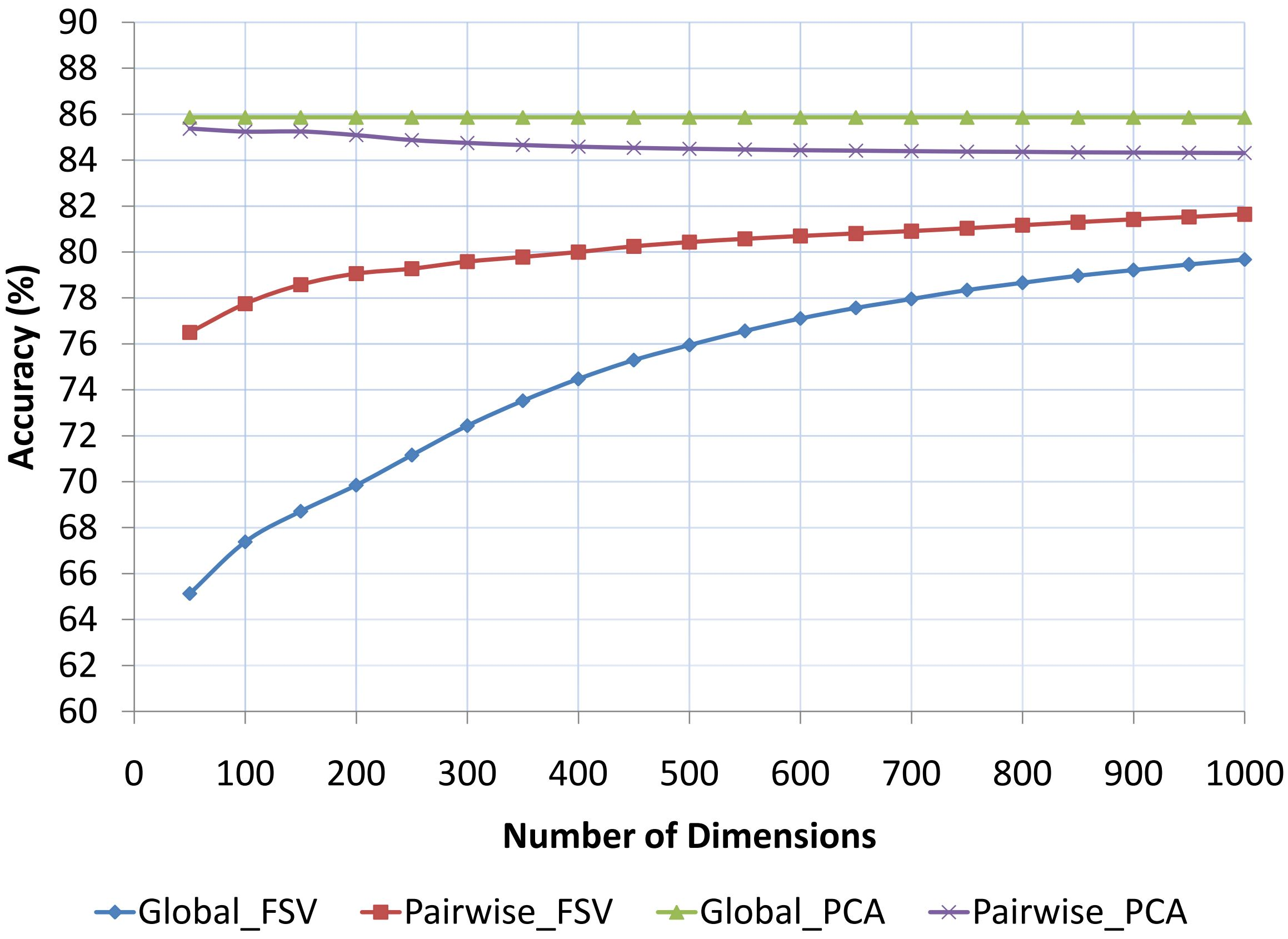

Supplement: S2 Fig — Comparison results are presented using two approaches: PCA and feature selection via concave minimization. Note that this experiment is performed using LPQ features with weak cross validation because of their low classification error. (TIF) [file pone.0197431.s002.tif]

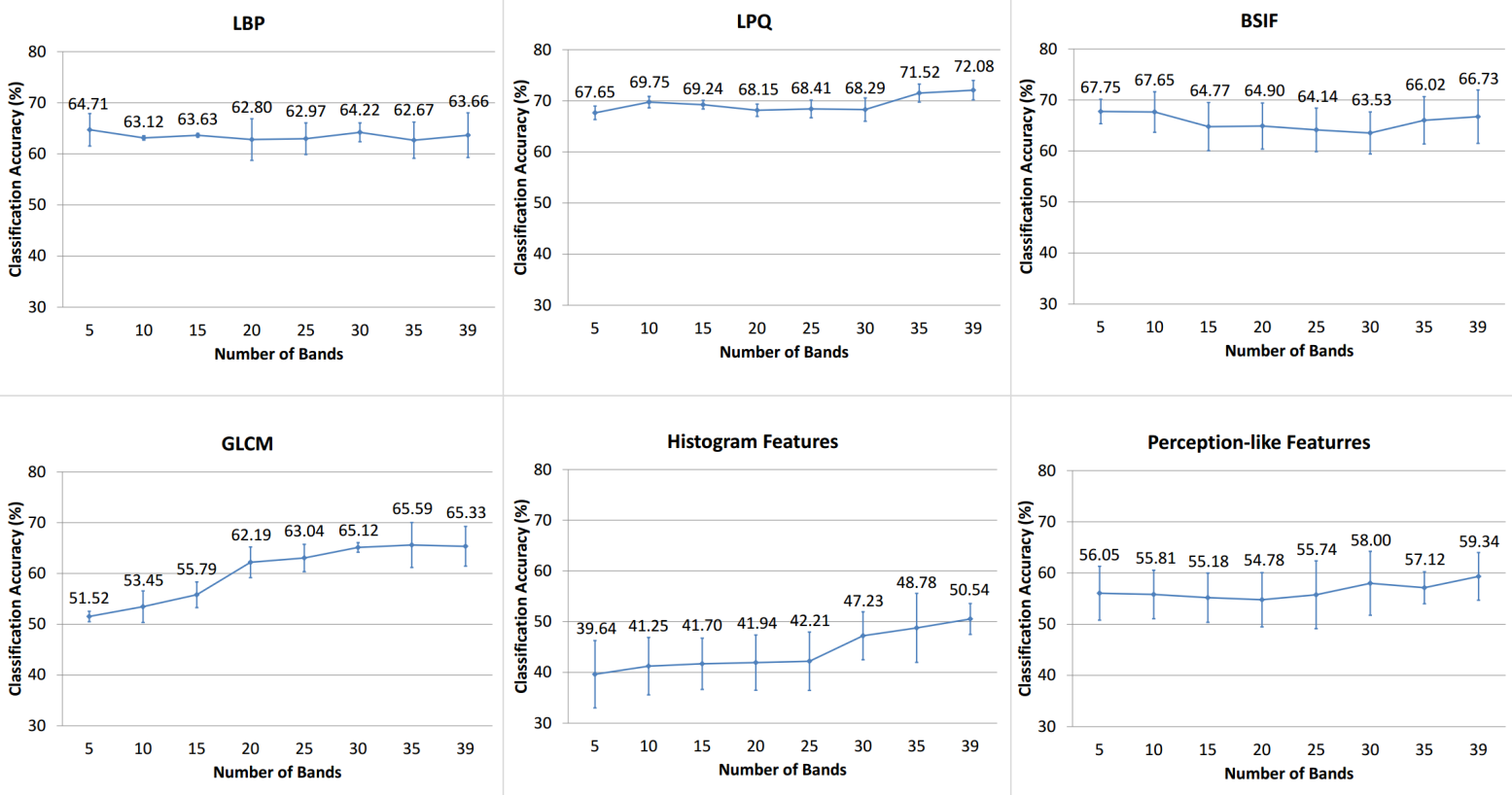

Supplement: S3 Fig — These results are generated by selecting varying number of bands for each texture descriptor. The error bars are generated as a result of cross validation. (PNG) [file pone.0197431.s003.png]
